# Prevalence of anxiety and depression and diagnostic utility of the CAT scale in spirometry-confirmed COPD patients: Findings from the Chinese Population Health and Multimorbidities Study

**DOI:** 10.1016/j.pccm.2026.08.003

**Published:** 2026-09-07

**Authors:** Sinan Wu, Jian Kang, Xiangyan Zhang, Lan Yang, Fuqiang Wen, Yuanlin Song, Jianping Zhao, Jianying Xu, Wen Li, Pixin Ran, Kewu Huang, Yahong Chen, Yanfei Guo, Guangliang Shan, Ting Yang, Chen Wang, Jieping Lei, Chinese Population Health and Multimorbidities Study (CPHMS) Investigators

**Affiliations:** a National Center for Respiratory Medicine; State Key Laboratory of Respiratory Health and Multimorbidity; National Clinical Research Center for Respiratory Diseases; Institute of Respiratory Medicine, Chinese Academy of Medical Sciences & Peking Union Medical College; Department of Clinical Research and Data Management, Center of Respiratory Medicine, China-Japan Friendship Hospital, Beijing 100029, China; b Department of Pulmonary and Critical Care Medicine, The First Hospital of China Medical University, Shenyang, Liaoning 110002, China; c Department of Pulmonary and Critical Care Medicine, Guizhou Provincial People’s Hospital, Guiyang, Guizhou 550001, China; d Department of Pulmonary and Critical Care Medicine, The First Affiliated Hospital of Xi’an Jiaotong University, Xi’an, Shaanxi 710061, China; e Department of Pulmonary and Critical Care Medicine, West China Hospital of Sichuan University, Chengdu, Sichuan 610044, China; f Department of Pulmonary and Critical Care Medicine, Zhongshan Hospital, Fudan University, Shanghai 200032, China; g Department of Pulmonary and Critical Care Medicine, Tongji Hospital, Tongji Medical College, Huazhong University of Science and Technology, Wuhan, Hubei 430010, China; h Department of Pulmonary and Critical Care Medicine, Shanxi Bethune Hospital, Shanxi Academy of Medical Sciences, Taiyuan, Shanxi 030032, China; i Department of Pulmonary and Critical Care Medicine, The Second Affiliated Hospital, School of Medicine, Zhejiang University, Hangzhou, Zhejiang 310009, China; j National Center for Respiratory Medicine; State Key Laboratory of Respiratory Disease; National Clinical Research Center for Respiratory Diseases; Guangzhou Institute of Respiratory Diseases; Department of Pulmonary and Critical Care Medicine, The First Affiliated Hospital, Guangzhou Medical University, Guangzhou, Guangdong 510163, China; k Department of Pulmonary and Critical Care Medicine, Beijing Chao-Yang Hospital, Capital Medical University, Beijing 100020, China; l Department of Pulmonary and Critical Care Medicine, Peking University Third Hospital, Beijing 100191, China; m National Center for Gerontology; Institute of Geriatric Medicine, Chinese Academy of Medical Sciences & Peking Union Medical College; Department of Pulmonary and Critical Care Medicine, Beijing Hospital, Beijing 100730, China; n Department of Epidemiology and Biostatistics, School of Basic Medicine, Peking Union Medical College; Institute of Basic Medical Sciences, Chinese Academy of Medical Sciences, Beijing 100730, China; o National Center for Respiratory Medicine; State Key Laboratory of Respiratory Health and Multimorbidity; National Clinical Research Center for Respiratory Diseases; Institute of Respiratory Medicine, Chinese Academy of Medical Sciences & Peking Union Medical College; Department of Pulmonary and Critical Care Medicine, Center of Respiratory Medicine, China-Japan Friendship Hospital, Beijing 100029, China; p School of Population Medicine and Public Health, Chinese Academy of Medical Sciences & Peking Union Medical College, Beijing 100730, China

**Keywords:** Prevalence, Anxiety, Depression, COPD Assessment Test (CAT), Chronic obstructive pulmonary disease, Multimorbidity

## Abstract

**Background:**

Patients with chronic obstructive pulmonary disease (COPD) frequently present with psychological comorbidities, including anxiety and depression, which may contribute to poorer clinical outcomes. However, the prevalence and impact of these mental health conditions in COPD patients have long been underrecognized. Leveraging data from the Chinese Population Health and Multimorbidities Study (CPHMS), a large epidemiological survey on chronic respiratory diseases in China, this study aimed to estimate the prevalence of anxiety and depression comorbidities among community-dwelling COPD patients and to evaluate the diagnostic utility of the COPD Assessment Test (CAT) scale for detecting these conditions in spirometry-confirmed COPD patients.

**Methods:**

Analytical data were derived from the CPHMS. Comprehensive information on sociodemographic characteristics, lifestyle factors, and health status was collected. COPD-related pulmonary and extra-pulmonary symptoms were assessed using the CAT scale, and anxiety and depression were evaluated via the Hospital Anxiety and Depression Scale. Multivariable logistic regression was employed to identify factors associated with anxiety or depression comorbidities, and receiver operating characteristic (ROC) curve analysis was used to assess the diagnostic performance of the CAT scale.

**Results:**

Among 3641 spirometry-confirmed community COPD patients, the prevalence of anxiety and depression was 11.07% (95% confidence interval [CI]: 10.05–12.09%) and 14.23% (95% CI: 13.09–15.36%), respectively. Female gender, rural residence, unemployment, lower annual family income, and higher total CAT score, as well as higher scores on both the pulmonary and extra-pulmonary symptom subscales of the CAT, were independently associated with an increased likelihood of anxiety and/or depression in COPD patients. The diagnostic yield of the CAT scale for identifying anxiety and depression in COPD patients was evaluated. In the overall COPD patients, the optimal total CAT cut-off value for detecting anxiety was 9 (area under the ROC curve [AUC]: 0.637; 95% CI: 0.607–0.666), and for depression was 7 (AUC: 0.633; 95% CI: 0.607–0.659). Notably, among rural-dwelling COPD patients, the optimal cut-off was 7 for both anxiety (AUC: 0.624; 95% CI: 0.582–0.665) and depression (AUC: 0.616; 95% CI: 0.579–0.653), whereas in urban patients, the corresponding cut-offs were 11 for anxiety (AUC: 0.652; 95% CI: 0.610–0.694) and 10 for depression (AUC: 0.654; 95% CI: 0.618–0.691).

**Conclusions:**

This study delineated the prevalence of anxiety and depression among community COPD patients and validated the clinical utility of the CAT scale in detecting these psychological comorbidities. Our findings underscore the pressing need for tailored psychological support, particularly for COPD patients residing in rural area of China.

## Introduction

Chronic obstructive pulmonary disease (COPD) is the most prevalent chronic respiratory disease (CRD) and the third leading cause of deaths in China, affecting over 99.9 million individuals nationwide.^1^^,^^2^ Beyond somatic comorbidities, such as circulatory, non-COPD respiratory, infectious, and digestive disorders, COPD patients frequently experience psychological comorbidities, including anxiety and depression.^3^, ^4^, ^5^, ^6^ These mental health conditions may contribute to adverse outcomes, such as increased disease complexity, reduced treatment adherence, acute respiratory exacerbation, hospitalization, diminished quality of life, disability, and even death.^7^, ^8^, ^9^, ^10^, ^11^, ^12^ Nevertheless, the prevalence and burden of anxiety and depression in COPD patients are often underestimated and, consequently, undertreated.^13^, ^14^, ^15^ Hence, identifying the characteristics of COPD patients with anxiety and/or depression is crucial for early detection and timely implementation of appropriate interventions.

According to the latest reports from the Global Initiative for Chronic Obstructive Lung Disease (GOLD), risk factors for anxiety and depression comorbidities in COPD patients include younger age, female gender, smoking, lower forced expiratory volume in one second (FEV_1_), cough, a higher St. George’s Respiratory Questionnaire score, and a history of cardiovascular disease.^3^^,^^16^, ^17^, ^18^ However, findings vary considerably across studies and settings, depending heavily on study design, population source, and the range of variables collected. Therefore, a reliable instrument is needed to assess the risk of psychological comorbidities in COPD patients.

The COPD Assessment Test (CAT) scale is a widely used simple tool for evaluating symptom burden and health status in COPD patients.^3^ It comprises eight items: the first four assess pulmonary symptoms (cough, phlegm/mucus, chest tightness, breathlessness), and the remaining four evaluate extra-pulmonary symptoms (activity, confidence, sleep, energy).^19^ Previous studies have reported a positive correlation between CAT scores and the likelihood of anxiety and/or depression comorbidities in COPD patients, as measured by the Hospital Anxiety and Depression Scale (HADS).^20^, ^21^, ^22^, ^23^ Moreover, several investigations have suggested that the extra-pulmonary component of the CAT scale may be superior to the pulmonary component in detecting psychological comorbidities in COPD patients.^24^^,^^25^ If this screening capability can be validated, it could enhance the diagnosis and management of psychological comorbidities in this population.

Most existing research on COPD patients with comorbidities has been conducted in hospital or clinical settings, whereas real-world data from community or general populations remain scarce. This study leverages data from the Chinese Population Health and Multimorbidities Study (CPHMS; originally named China Pulmonary Health Study, CPHS),^1^^,^^26^ a large population-based epidemiological survey on CRDs in China. We aimed to estimate the prevalence of comorbid anxiety and depression, to investigate the association between CAT scores—including its pulmonary and extra-pulmonary subscales—and these psychological comorbidities, and to evaluate the diagnostic utility of the CAT scale for identifying psychological disorders among community-dwelling COPD patients.

## Methods

### Ethics approval and consent to participate

This study involving human participants adhered to the *Declaration of Helsinki*, and was reviewed and approved by the Institutional Review Board of the Beijing Chao-Yang Hospital (approval number: 11-KE-42). The participants provided their written informed consent to participate in this study.

### Study design

Individual-level data for this study were derived from the CPHMS. The CPHMS employed a multistage stratified cluster random sampling design and enrolled 50,991 participants aged 20 years or older from 10 provinces and municipalities spanning seven geographical and administrative regions during 2012–2015. The survey population is broadly representative of the general Chinese adult population, covering both genders and urban as well as rural areas. Detailed descriptions of the CPHMS design have been published previously.^1^^,^^26^

### Measurements

Data on sociodemographic characteristics, potential associated factors, respiratory symptoms, comorbidities, CAT and HADS assessments were collected through a comprehensive questionnaire. Anthropometric measurements (height and weight), as well as pre- and post-bronchodilator pulmonary function tests (with the use of spirometer) were conducted by trained physicians, nurses, and/or technicians. Exposure to ambient particulate matter with an aerodynamic diameter ≤2.5 µm (PM_2.5_) was estimated using a regional satellite‑based aerosol optical depth model.^27^ Annual mean PM_2.5_ concentrations for 2010 were assigned to each participant based on their urban district or rural county of residence.

The CAT scale comprises eight items: the first four assess pulmonary symptoms (cough, phlegm/mucus, chest tightness, and breathlessness), and the remaining four evaluate extra‑pulmonary symptoms (activity, confidence, sleep, and energy).^19^^,^^28^ Each item is scored from 0 (with no symptom) to 5 (with most severe symptom), reflecting symptom severity. The total CAT score ranges from 0 to 40, with higher scores indicating greater symptom burden and worse health status.^19^^,^^28^

Anxiety and depression were assessed using the HADS, a validated instrument for screening these two psychological conditions.^29^, ^30^, ^31^ The HADS consists of 14 items, with seven items for the anxiety subscale and seven for the depression subscale.^29^, ^30^, ^31^ Each item is scored from 0 (with no symptom) to 3 (with most severe symptom), yielding subscale scores from 0 to 21; scores of 0–7 are classified as non‑cases, 8–10 as possible cases, and 11–21 as probable cases.^29^, ^30^, ^31^ A cut‑off of ≥ 8 was used to identify possible or probable anxiety or depression.^29^, ^30^, ^31^ The HADS has been validated for use in the Chinese population.^32^

### Study population

COPD was defined according to the GOLD criteria as a post‑bronchodilator FEV_1_/forced vital capacity (FVC) ratio < 0.70.^3^ Of the 4908 spirometry‑confirmed COPD patients, 1267 were excluded due to missing CAT and/or HADS data, leaving 3641 participants for the final analysis. The study flowchart is presented in Fig. 1, and a comparison between included and excluded individuals is provided in Supplementary Table 1.

**Fig. 1:**
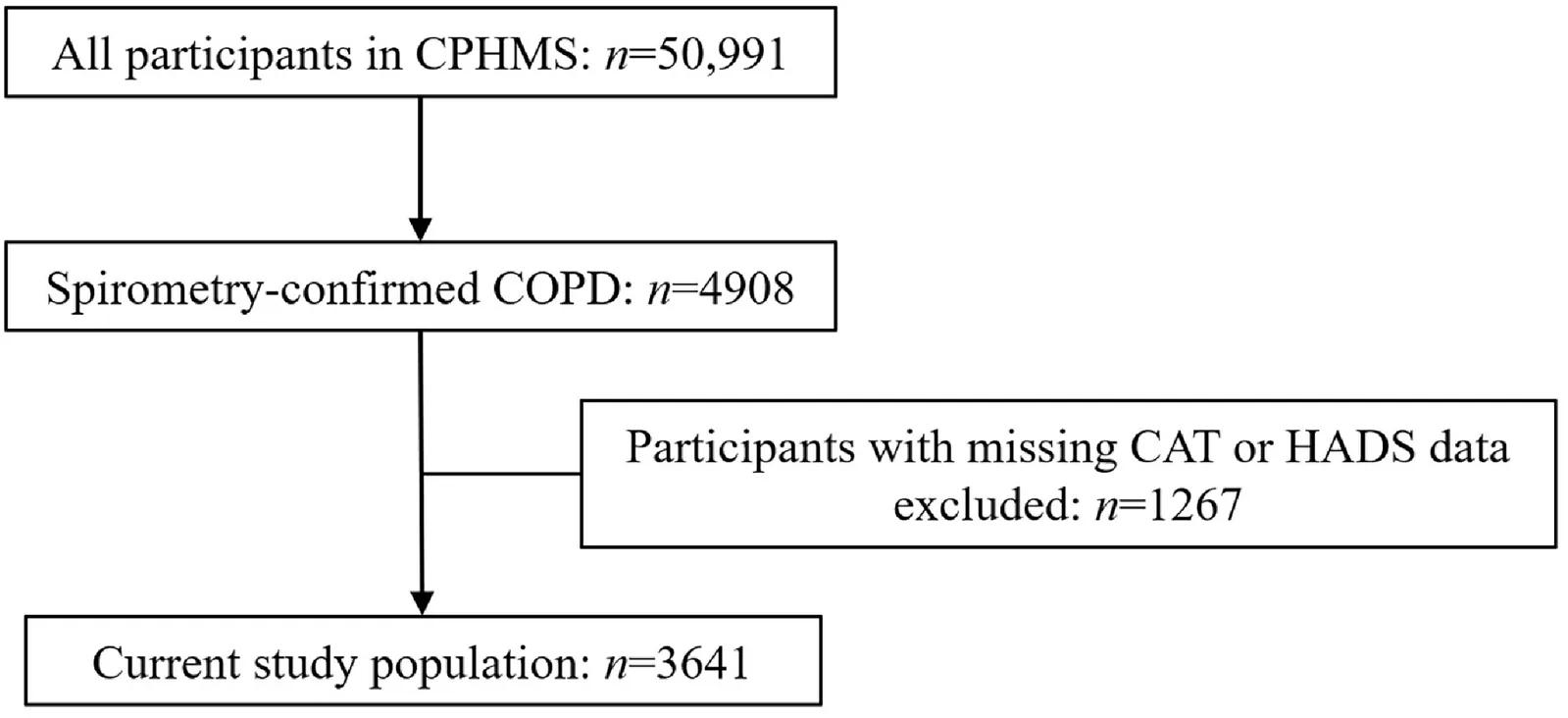
Study flowchart for the inclusion of 3641 COPD patients in this study. CAT, Chronic Obstructive Pulmonary Disease Assessment Test; COPD, Chronic obstructive pulmonary disease; CPHMS, Chinese Population Health and Multimorbidity Study; HADS, Hospital Anxiety and Depression Scale.

### Statistical analyses

Continuous variables with skewed distribution were expressed as medians with interquartile ranges, and group comparisons were performed using the Mann-Whitney *U* test. Categorical variables were summarized as counts and percentages and compared using the Chi‑square test or Fisher’s exact test, as appropriate. Shapiro-Wilk test (when *n* ≤ 2000) or Kolmogorov-Smirnov test (when *n* > 2000) was used for normality test.

Three multivariable logistic regression models were constructed to identify factors associated with the prevalence of anxiety or depression among COPD patients. In Model 1, the dependent variable was the presence of anxiety (yes/no) or depression (yes/no), and the 16 adjustment variables were: age (years), gender (male/female), BMI (kg/m²), residence (urban/rural), annual mean PM_2.5_ exposure (<50/50–74/≥75 µg/m³), educational level (primary school or below/junior high school or above), employment status (employed/unemployed), annual total family income (ten thousand yuan), smoking index (pack‑years), comorbidities (coronary heart disease, stroke, hypertension, diabetes, and cancer; each yes/no), CAT total score (points), and GOLD stage (Ⅰ/Ⅱ/Ⅲ–Ⅳ). Sensitivity analyses were performed by excluding the sixth (confidence) and eighth (energy) items from the CAT total score.

In Model 2, the CAT total score was replaced by the CAT Q1234 score (points) and Q5678 score (points). In Model 3, the CAT total score (points) and a binary indicator of whether the CAT Q5678 score was greater than or equal to the Q1234 score (yes/no) were used.

Model performance was evaluated using events per variable, receiver operating characteristic (ROC) curve, area under the ROC curve (AUC), Hosmer-Lemeshow goodness‑of‑fit test, internal validation in a randomly split training set (70%) and validation set (30%), calibration plot, and multicollinearity diagnostic.

ROC curve, AUC, sensitivity, specificity, and Youden’s index were used to determine the optimal CAT cut‑off values for detecting anxiety or depression in COPD patients. All statistical analyses were performed using SAS version 9.4 (SAS Institute, Cary, NC, USA) and the pROC and ggplot2 packages in R (https://www.r-project.org/). All tests were two‑sided, and a *P* value < 0.05 was considered statistically significant.

## Results

### Prevalence and characteristics of COPD patients comorbid with anxiety and depression

Among the 3641 spirometry-confirmed COPD patients, 1952 (53.61%), 1376 (37.79%) and 313 (8.60%) were classified as GOLD stages Ⅰ, Ⅱ, and Ⅲ–Ⅳ, respectively. The prevalence of anxiety and depression, as determined by the HADS, was 11.07% (403/3641; 95% confidence interval [CI]: 10.05–12.09%) and 14.23% (518/3641; 95% CI: 13.09–15.36%), respectively (Supplementary Table 1 and Table 1).

**Table 1:** Comparisons of COPD patients comorbid with anxiety versus without anxiety, and comorbid with depression versus without depression, respectively.

Variables	Anxiety (*n* = 403)	No anxiety (*n* = 3238)	Statistics	*P* values	Depression (*n* = 518)	No depression (*n* = 3123)	Statistics	*P* values
Age (years), median (IQR)	61 (54, 68)	61 (54, 68)	742,475.50^a^	0.665	63 (56, 70)	61 (53, 68)	1,036,221.50^a^	<0.001
Female, *n* (%)	177 (43.92)	1150 (35.52)	10.93^b^	0.001	216 (41.70)	1111 (35.57)	7.19^b^	0.007
BMI (kg/m^2^), median (IQR)	23.31 (20.54, 25.91)	23.62 (21.48, 25.89)	695,008.50^a^	0.051	23.24 (20.57, 25.83)	23.66 (21.48, 25.91)	891,364.50^a^	0.019
Rural residents, *n* (%)	210 (52.11)	1236 (38.17)	29.08^b^	<0.001	271 (52.32)	1175 (37.62)	40.06^b^	<0.001
Annual mean PM_2.5_ exposure, *n* (%)			13.66^b^	0.001			12.24^b^	0.002
<50 μg/m^3^	31 (7.69)	193 (5.96)			38 (7.34)	186 (5.96)		
50–74 μg/m^3^	278 (68.98)	2003 (61.86)			352 (67.95)	1929 (61.77)		
≥75 μg/m^3^	94 (23.33)	1042 (32.18)			128 (24.71)	1008 (32.28)		
Junior high school or above, *n* (%)	197 (48.88)	1866 (57.63)	11.16^b^	0.001	233 (44.98)	1830 (58.60)	33.55^b^	<0.001
Currently married, *n* (%)	361 (89.58)	2914 (89.99)	0.07^b^	0.794	457 (88.22)	2818 (90.23)	1.98^b^	0.159
Currently employed, *n* (%)	272 (67.49)	2438 (75.29)	11.46^b^	0.001	352 (67.95)	2358 (75.50)	13.31^b^	<0.001
Annual family income (ten thousand yuan), median (IQR)	2.00 (1.00, 3.00)	2.40 (1.00, 5.00)	604,491.00^a^	<0.001	2.00 (1.00, 3.00)	2.50 (1.00, 5.00)	802,371.00^a^	<0.001
Smoking status, *n* (%)			3.90^b^	0.142			5.36^b^	0.069
Never smoker	208 (51.61)	1562 (48.24)			276 (53.28)	1494 (47.84)		
Ex-smoker	41 (10.17)	438 (13.53)			60 (11.58)	419 (13.42)		
Current smoker	154 (38.21)	1238 (38.23)			182 (35.14)	1210 (38.74)		
Smoking index (pack-years), median (IQR)	0 (0, 27.00)	0 (0, 31.00)	630,245.50^a^	0.100	0 (0, 29.50)	0.80 (0, 31.00)	820,527.00^a^	0.044
Respiratory symptom, *n* (%)								
Cough	103 (25.56)	512 (15.81)	24.49^b^	<0.001	109 (21.04)	506 (16.20)	7.35^b^	0.007
Sputum	131 (32.51)	693 (21.40)	25.19^b^	<0.001	145 (27.99)	679 (21.74)	9.82^b^	0.002
Wheeze	122 (30.27)	564 (17.42)	39.38^b^	<0.001	145 (27.99)	541 (17.32)	33.36^b^	<0.001
Dyspnea	129 (32.01)	565 (17.45)	48.89^b^	<0.001	145 (27.99)	549 (17.58)	30.19^b^	<0.001
Comorbidity, *n* (%)								
Coronary heart disease	19 (4.71)	94 (2.90)	2.81^b^	0.093	26 (5.02)	87 (2.79)	5.36^b^	0.021
Stroke	7 (1.74)	38 (1.17)	0.61^b^	0.434	12 (2.32)	33 (1.06)	4.54^b^	0.033
Hypertension	46 (11.41)	396 (12.23)	1.00^b^	0.318	65 (12.55)	377 (12.07)	0.15^b^	0.697
Diabetes	11 (2.73)	123 (3.80)	1.76^b^	0.185	18 (3.47)	116 (3.71)	0.39^b^	0.534
Malignancy	1 (0.25)	5 (0.15)	NA^c^	0.530	0 (0)	6 (0.19)	NA^c^	0.599
CAT								
CAT total score (points), median (IQR)	9 (4, 16)	5 (2, 10)	911,930.50^a^	<0.001	9 (4, 14)	5 (2, 10)	1,158,664.50^a^	<0.001
CAT total score ≥ 10, *n* (%)	187 (46.40)	854 (26.37)	70.41^b^	<0.001	236 (45.56)	805 (25.78)	85.17^b^	<0.001
CAT total score categories, *n* (%)			91.97^b^	<0.001			97.17^b^	<0.001
0–9	216 (53.60)	2384 (73.63)			282 (54.44)	2318 (74.22)		
10–19	118 (29.28)	653 (20.17)			161 (31.08)	610 (19.53)		
20–29	59 (14.64)	175 (5.40)			61 (11.78)	173 (5.54)		
30–40	10 (2.48)	26 (0.80)			14 (2.70)	22 (0.70)		
CAT Q1234 score (points), median (IQR)	5 (2, 9)	3 (1, 6)	891,019.50^a^	<0.001	5 (2, 8)	3 (1, 6)	1,125,517.50^a^	<0.001
CAT Q5678 score (points), median (IQR)	4 (1, 8)	2 (0, 4)	904,320.00^a^	<0.001	4 (1, 7)	2 (0, 4)	1,154,029.50^a^	<0.001
Lung function indices, median (IQR)								
FEV_1_ (L)	1.88 (1.37, 2.39)	2.07 (1.61, 2.58)	636,567.50^a^	<0.001	1.84 (1.38, 2.36)	2.08 (1.62, 2.59)	791,959.00^a^	<0.001
FEV_1_%pred (%)	77.93 (60.87, 92.16)	82.47 (68.39, 94.52)	658,681.00^a^	<0.001	77.95 (60.87, 92.67)	82.58 (68.43, 94.52)	857,462.50^a^	<0.001
FEV_1_/FVC (%)	62.99 (51.82, 67.48)	64.13 (56.81, 67.55)	679,654.50^a^	0.007	62.71 (54.18, 67.33)	64.20 (56.92, 67.62)	872,008.50^a^	0.001
GOLD stage, *n* (%)			20.72^b^	<0.001			28.83^b^	<0.001
GOLD Ⅰ	192 (47.64)	1760 (54.35)			245 (47.30)	1707 (54.66)		
GOLD Ⅱ	153 (37.97)	1223 (37.77)			198 (38.22)	1178 (37.72)		
GOLD Ⅲ–Ⅳ	58 (14.39)	255 (7.88)			75 (14.48)	238 (7.62)		

BMI, Body mass index; CAT, Chronic Obstructive Pulmonary Disease Assessment Test; COPD, Chronic obstructive pulmonary disease; FEV_1_, Forced expiratory volume in one second; FEV_1_%pred, Measured forced expiratory volume in one second expressed as a percentage of the predicted value; FVC, Forced vital capacity; GOLD, Global Initiative for Chronic Obstructive Lung Disease; IQR, Interquartile range; PM_2.5_, Particulate matter with a diameter <2.5 μm; SD, Standard deviation. CAT Q1234 are the first four questions in the CAT scale, assessing pulmonary symptoms (cough, phlegm/mucus, chest tightness, and breathlessness).CAT Q5678 are the remaining four questions in the CAT scale, assessing extra-pulmonary symptoms (activity, confidence, sleep, and energy).

a *U* value (Mann-Whitney *U* test for continuous variable).

b *χ^2^* value (Chi-squared test for categorical variable).

c “NA” (not applicable), indicating Fisher’s exact test was applied and specific statistic was not applicable.

Compared with COPD patients without anxiety, those with anxiety were more likely to be female, reside in rural areas, have lower exposure to ambient particulate matter, have lower educational attainment (primary school or below), be unemployed, and have lower annual family income. In addition, anxious patients reported more pulmonary symptoms (cough, sputum production, wheeze, and dyspnea), had higher CAT scores, and exhibited worse pulmonary function (Table 1).

Patients with depression showed similar features. Notably, compared with those without depression, depressed patients were older, had lower smoking exposure, and had a higher prevalence of comorbidity with coronary heart disease and stroke (Table 1).

### Differences between pulmonary- and extra-pulmonary-dominant CAT subscale groups

Among the study population, 55.78% (2031/3641; 95% CI: 54.17–57.39%) had pulmonary-dominant symptoms (Q1234 > Q5678), while 44.22% (1610/3641; 95% CI: 42.61–45.83%) had extra-pulmonary-dominant symptoms (Q5678 ≥ Q1234). Compared with the pulmonary-dominant group, patients with extra-pulmonary dominance were more often female, less likely to be married, had fewer current smokers, with a lower smoking index, had a lower total CAT score, and had better pulmonary function (Supplementary Table 2).

The prevalence of anxiety in the pulmonary-dominant and extra-pulmonary-dominant groups was 11.37% (231/2031; 95% CI: 9.99–12.75%) and 10.68% (172/1610; 95% CI: 9.17–12.19%), respectively; the corresponding prevalence of depression was 14.28% (290/2031; 95% CI: 12.76–15.80%) and 14.16% (228/1610; 95% CI: 12.46–15.86%) (Supplementary Table 2).

### Associations between the CAT scale and anxiety and depression in COPD patients

In the fully adjusted regression model, female gender (OR: 1.355; 95% CI: 1.017–1.806; *P* = 0.038), rural residence (OR: 1.647; 95% CI: 1.269–2.137; *P* < 0.001), and a higher CAT total score (OR: 1.063; 95% CI: 1.046–1.080; *P* < 0.001) were independently associated with increased prevalence of anxiety, whereas current employment (OR: 0.691; 95% CI: 0.527–0.907; *P* = 0.008) and higher annual family income (OR: 0.910; 95% CI: 0.860–0.963; *P* = 0.001) were associated with lower prevalence (Table 2).

**Table 2:** Associations of CAT total score and other influencing factors with comorbidity of anxiety or depression in COPD patients, respectively.

Associated factors	Anxiety		Depression	
	OR (95% CI)	*P* value	OR (95% CI)	*P* value
Age	0.988 (0.977–1.000)	0.059	1.000 (0.989–1.011)	0.981
Female	1.355 (1.017–1.806)	0.038	1.103 (0.853–1.425)	0.455
BMI	0.995 (0.960–1.030)	0.770	0.989 (0.958–1.021)	0.483
Rural residence	1.647 (1.269–2.137)	<0.001	1.768 (1.400–2.234)	<0.001
Annual mean PM_2.5_ exposure				
<50 μg/m^3^	Ref.		Ref.	
50–74 μg/m^3^	1.419 (0.869–2.318)	0.162	1.235 (0.798–1.912)	0.343
≥75 μg/m^3^	1.048 (0.611–1.799)	0.864	1.002 (0.622–1.616)	0.993
Junior high school or above	1.006 (0.764–1.323)	0.969	0.823 (0.644–1.051)	0.119
Currently employed	0.691 (0.527–0.907)	0.008	0.771 (0.603–0.985)	0.038
Annual family income	0.910 (0.860–0.963)	0.001	0.959 (0.919–1.001)	0.058
Smoking index	1.000 (0.994–1.006)	0.988	0.994 (0.988–0.999)	0.033
Coronary heart disease	1.191 (0.670–2.116)	0.552	1.203 (0.718–2.015)	0.483
Stroke	1.064 (0.423–2.677)	0.895	1.867 (0.904–3.858)	0.092
Hypertension	0.852 (0.590–1.230)	0.392	0.857 (0.622–1.181)	0.347
Diabetes	0.718 (0.370–1.395)	0.328	0.888 (0.515–1.529)	0.667
Malignancy	0.972 (0.108–8.758)	0.980	NA	NA
CAT total score	1.063 (1.046–1.080)	<0.001	1.056 (1.040–1.072)	<0.001
GOLD stage				
GOLD Ⅰ	Ref.		Ref.	
GOLD Ⅱ	0.925 (0.710–1.205)	0.564	0.960 (0.758–1.217)	0.738
GOLD Ⅲ–Ⅳ	1.112 (0.747–1.655)	0.601	1.218 (0.852–1.741)	0.280

BMI, Body mass index; CAT, Chronic Obstructive Pulmonary Disease Assessment Test; CI, Confidence interval; COPD, Chronic obstructive pulmonary disease; GOLD, Global Initiative for Chronic Obstructive Lung Disease; NA, Not applicable; OR, Odds Ratio; PM_2.5_, Particulate matter with a diameter <2.5 μm; Ref., Reference group.

For depression, rural residence (OR: 1.768; 95% CI: 1.400–2.234; *P* < 0.001) and a higher CAT total score (OR: 1.056; 95% CI: 1.040–1.072; *P* < 0.001) were associated with higher prevalence, while current employment (OR: 0.771; 95% CI: 0.603–0.985; *P* = 0.038) was associated with lower prevalence (Table 2). Sensitivity analyses, excluding the sixth (confidence) and eighth (energy) items from the CAT total score, yielded consistent findings (Supplementary Table 3).

When both CAT Q1234 and Q5678 scores were included in the same regression model, both subscale scores remained significantly and independently associated with increased prevalence of anxiety (Q1234: OR = 1.055; 95% CI: 1.015–1.096; *P* = 0.007; Q5678: OR = 1.071; 95% CI: 1.028–1.117; *P* = 0.001) and depression (Q1234: OR = 1.037; 95% CI: 1.001–1.074; *P* = 0.044; Q5678: OR = 1.077; 95% CI: 1.037–1.119; *P*  <  0.001) (Table 3).

**Table 3:** Associations of CAT pulmonary and extra-pulmonary component scores, and other influencing factors with comorbidity of anxiety or depression in COPD patients, respectively.

Associated factors	Anxiety		Depression	
	OR (95% CI)	*P* value	OR (95% CI)	*P* value
Age	0.988 (0.976–1.000)	0.057	1.000 (0.989–1.011)	0.985
Female	1.353 (1.015–1.803)	0.039	1.097 (0.849–1.418)	0.478
BMI	0.995 (0.961–1.031)	0.788	0.990 (0.959–1.022)	0.520
Rural residence	1.649 (1.271–2.141)	<0.001	1.775 (1.405–2.242)	<0.001
Annual mean PM_2.5_ exposure				
<50 μg/m^3^	Ref.		Ref.	
50–74 μg/m^3^	1.431 (0.874–2.342)	0.154	1.259 (0.812–1.951)	0.304
≥75 μg/m^3^	1.060 (0.616–1.824)	0.834	1.027 (0.635–1.661)	0.913
Junior high school or above	1.007 (0.765–1.326)	0.959	0.826 (0.646–1.055)	0.126
Currently employed	0.694 (0.528–0.911)	0.009	0.778 (0.608–0.995)	0.045
Annual family income	0.910 (0.860–0.963)	0.001	0.959 (0.919–1.002)	0.059
Smoking index	1.000 (0.994–1.006)	0.989	0.994 (0.989–1.000)	0.039
Coronary heart disease	1.188 (0.668–2.112)	0.558	1.197 (0.714–2.007)	0.496
Stroke	1.048 (0.415–2.647)	0.922	1.811 (0.873–3.760)	0.111
Hypertension	0.850 (0.589–1.228)	0.387	0.854 (0.619–1.176)	0.333
Diabetes	0.718 (0.370–1.394)	0.328	0.888 (0.515–1.530)	0.668
Malignancy	0.981 (0.109–8.836)	0.986	NA	NA
CAT Q1234 score	1.055 (1.015–1.096)	0.007	1.037 (1.001–1.074)	0.044
CAT Q5678 score	1.071 (1.028–1.117)	0.001	1.077 (1.037–1.119)	<0.001
GOLD stage				
GOLD Ⅰ	Ref.		Ref.	
GOLD Ⅱ	0.931 (0.713–1.216)	0.600	0.975 (0.768–1.238)	0.836
GOLD Ⅲ–Ⅳ	1.122 (0.752–1.674)	0.573	1.243 (0.868–1.780)	0.236

BMI, Body mass index; CAT, Chronic Obstructive Pulmonary Disease Assessment Test; CI, Confidence interval; COPD, Chronic obstructive pulmonary disease; GOLD, Global Initiative for Chronic Obstructive Lung Disease; NA, Not applicable; OR, Odds ratio; PM_2.5_, Particulate matter with a diameter <2.5 μm; Ref., Reference group. CAT Q1234 are the first four questions in the CAT scale, assessing pulmonary symptoms (cough, phlegm/mucus, chest tightness, and breathlessness). CAT Q5678 are the remaining four questions in the CAT scale, assessing extra-pulmonary symptoms (activity, confidence, sleep, and energy).

However, when the CAT total score and the dichotomous variable indicating whether Q5678 was ≥ Q1234 were entered together in the same model, no significant association was observed for this dichotomous variable with either anxiety or depression (Supplementary Table 4).

All multivariable regression models demonstrated good fit, as assessed by multiple diagnostic methods (detailed in Supplementary File).

### Diagnostic performance of the CAT scale for anxiety and depression in COPD patients

ROC analysis identified the optimal CAT total score cut-off for detecting anxiety in the overall COPD population as 9 (AUC: 0.637; 95% CI: 0.607–0.666) (Supplementary Table 5 and Table 4). Stratified by residence, the optimal cut-offs were 7 (AUC: 0.624; 95% CI: 0.582–0.665) for rural patients and 11 (AUC: 0.652; 95% CI: 0.610–0.694) for urban patients (Supplementary Table 6 and Table 4). For the subscales, the optimal cut-offs for anxiety were 5 for Q1234 (AUC: 0.620; 95% CI: 0.591–0.650) and 4 for Q5678 (AUC: 0.631; 95% CI: 0.601–0.660) in all patients (Supplementary Table 7 and Table 4).

**Table 4:** The optimal cut-off values of CAT scores for anxiety or depression assessment in COPD patients, respectively.

Psychological disease assessment	Study population	Optimal cut-off point	AUC (95% CI)	Specificity (%)	Sensitivity (%)	Youden index
Anxiety	All COPD patients	CAT total score: 9	0.637 (0.607–0.666)	69.117	51.861	0.210
	All COPD patients	CAT Q1234 score: 5	0.620 (0.591–0.650)	63.650	53.846	0.175
	All COPD patients	CAT Q5678 score: 4	0.631 (0.601–0.660)	68.159	52.109	0.203
	COPD patients living in rural area	CAT total score: 7	0.624 (0.582–0.665)	58.900	61.429	0.203
	COPD patients living in urban area	CAT total score: 11	0.652 (0.610–0.694)	77.522	46.114	0.236
Depression	All COPD patients	CAT total score: 7	0.633 (0.607–0.659)	58.501	62.741	0.212
	All COPD patients	CAT Q1234 score: 5	0.613 (0.587–0.639)	64.233	53.475	0.177
	All COPD patients	CAT Q5678 score: 4	0.630 (0.604–0.656)	68.780	51.351	0.201
	COPD patients living in rural area	CAT total score: 7	0.616 (0.579–0.653)	59.745	60.517	0.203
	COPD patients living in urban area	CAT total score: 10	0.654 (0.618–0.691)	73.819	49.798	0.236

AUC, Area under the receiver operating characteristic curve; CAT, Chronic Obstructive Pulmonary Disease Assessment Test; CI, Confidence interval; COPD, Chronic obstructive pulmonary disease.

For depression, the optimal total CAT cut-off was 7 (AUC: 0.633; 95% CI: 0.607–0.659) in the overall population (Supplementary Table 8 and Table 4). Rural and urban patients had optimal cut-offs of 7 (AUC: 0.616; 95% CI: 0.579–0.653) and 10 (AUC: 0.654; 95% CI: 0.618–0.691), respectively (Supplementary Table 9 and Table 4). For the subscales, the optimal cut-offs for depression were 5 for Q1234 (AUC: 0.613; 95% CI: 0.587–0.639) and 4 for Q5678 (AUC: 0.630; 95% CI: 0.604–0.656) in all patients (Supplementary Table 10 and Table 4).

## Discussion

In this large-scale, population-based study in China, we estimated the prevalence and associated factors of anxiety and depression among COPD patients, and determined the optimal CAT score cut-offs for identifying these psychological comorbidities.

The observed prevalence of anxiety and depression was 11.07% and 14.23%, respectively, which is lower than that reported in many previous studies.^33^^,^^34^ Notably, excluded COPD patients exhibited milder airflow limitation compared to those included in the analytical sample (Supplementary Table 1), suggesting that the actual prevalence in community-dwelling COPD patients may be even lower than our current estimates. Given that our data were derived from the general population, the prevalence figures are understandably lower than those from hospital- or clinic-based studies.^35^ Psychological comorbidities in COPD are often underestimated and subsequently undertreated,^18^^,^^36^ partly because existing psychological assessment tools are complex and not easily applicable, particularly in primary care settings.

Prior research has consistently shown a positive correlation between CAT and HADS scores, indicating that higher CAT scores are associated with a greater likelihood of anxiety and/or depression in COPD patients.^20^, ^21^, ^22^, ^23^ Accordingly, we proposed that the CAT scale could serve as a feasible screening tool for early detection of these mental health conditions. A cross-sectional study reported that a higher total CAT score was predominantly driven by the extra-pulmonary component.^37^ Two additional studies—one cohort study conducted in Japan and Korea, and another cross-sectional study from China—both found that the extra-pulmonary component of the CAT scale outperformed the pulmonary component in identifying anxiety and depression.^24^^,^^25^ In our validation, although we confirmed the screening utility of the CAT scale, we did not observe superiority of the extra-pulmonary component. This discrepancy may be due to differences in overall symptom burden between community and clinical COPD populations. Extra-pulmonary symptoms as measured by the CAT scale may lack sufficient specificity to differentiate psychological distress in the general population. Moreover, heterogeneous findings across studies could reflect variations in research settings, cultural contexts, and the psychometric validity of the CAT scale in different regions. Nevertheless, we consider the CAT scale a brief and practical instrument capable of simultaneously capturing respiratory and psychological symptoms, making it well-suited for routine use in primary care.

The factors associated with coexisting anxiety and/or depression in COPD patients remain contentious,^38^^,^^39^ largely due to variability in the analytical variables included across studies. In our analysis, beyond CAT scores, sociodemographic factors—including female gender, rural residence, current unemployment, and lower family income—were independently associated with increased likelihood of anxiety and/or depression. These findings help delineate target populations that may benefit from tailored interventions. Given the cross-sectional design of this study, longitudinal investigations are warranted to validate our findings and to establish causal relationships. Systematic reviews and meta-analyses would also be valuable to synthesize evidence on potential influencing factors. Additionally, integrating comprehensive medical assessments—such as psychological evaluations and neuroimaging—could further elucidate the underlying mechanisms of psychological comorbidities in COPD.

The generally recommended CAT total score cut-off of 10 is used to indicate greater symptom burden and worse health status in COPD patients.^3^ In our study, the optimal cut-offs for identifying anxiety and depression were 9 and 7, respectively. Notably, among rural-dwelling COPD patients, the optimal cut-off was 7 for both conditions, whereas in urban patients, the cut-offs were 11 for anxiety and 10 for depression. These results suggest that rural COPD patients may be more susceptible to psychological distress, posing a public health, medical, and social challenge. The underlying reasons and contributing factors merit further exploration, and targeted interventions should be promptly developed for these high-risk groups. Although some integrated intervention models—combining physical, psychological, lifestyle modifications, pulmonary rehabilitation, and/or cognitive behavioral therapies—have been investigated,^40^, ^41^, ^42^, ^43^, ^44^ their effects have generally been modest, highlighting the need for more effective strategies.

Unlike studies that recruited participants from hospital or clinic settings, our study population and data—derived from the CPHMS—are highly representative of the general adult population in China. Our findings support the use of the CAT scale as a simple and practical tool for early detection of psychological comorbidities in COPD patients, suitable for routine application in primary care. Furthermore, we identified notable urban–rural differences in the optimal CAT cut-off values for detecting anxiety and depression, suggesting that rural patients may be more vulnerable to psychological issues. These findings can assist clinicians and researchers in more accurately identifying patients who would benefit from psychological support. Enhanced disease management policies and interventions should be prioritized for rural patients to mitigate disparities between urban and rural areas.

Despite providing valuable real-world data, this study has several limitations. First, the cross-sectional design precludes causal inferences regarding the relationship between CAT scores, associated factors, and the prevalence of anxiety and/or depression in COPD patients. Second, because participants were recruited from the general community, the applicability of our findings to outpatient and inpatient COPD populations remains uncertain.

To address these limitations, we propose three specific directions for future research: (1) conduct prospective cohort studies to examine the causal relationship between changes in CAT scores and the onset and progression of anxiety and/or depression in COPD patients; (2) incorporate additional clinical confounders—such as treatment regimens and history of acute exacerbations—to further validate the screening performance of the CAT scale; and (3) design intervention studies based on CAT-guided screening to evaluate the impact of early detection and management of psychological comorbidities on patient outcomes, including acute exacerbation rates, quality of life, and healthcare costs.

In conclusion, this study provided empirical estimates of the prevalence of anxiety and depression among community-dwelling COPD patients in China and validated the utility of the CAT scale as a screening tool for these psychological comorbidities. Our findings further underscore the heightened vulnerability of rural COPD patients to psychological distress, highlighting the urgent need for tailored mental health support and targeted interventions in underserved areas.

## Funding

This study was supported by the Science and Technology Fundamental Resources Investigation Program (No. 2023FY100602); Non-Profit Central Research Institute Fund of Chinese Academy of Medical Sciences (No. 2022-ZHCH330-01); National High Level Hospital Clinical Research Funding (No. 2025-NHLHCRF-DLYJ-PY-15); China International Medical Foundation (No. Z-2017-24-2301); and Chinese Academy of Medical Science Institute of Respiratory Medicine Grant for Young Scholars (No. 2023-ZF-9).

## Availability of data and materials

The data supporting the conclusions of this study will be made available from the corresponding authors on reasonable request.

## CRediT authorship contribution statement

**Sinan Wu:** Writing – original draft, Project administration, Funding acquisition, Conceptualization. **Jian Kang:** Investigation. **Xiangyan Zhang:** Investigation. **Lan Yang:** Investigation. **Fuqiang Wen:** Investigation. **Yuanlin Song:** Investigation. **Jianping Zhao:** Investigation. **Jianying Xu:** Investigation. **Wen Li:** Investigation. **Pixin Ran:** Investigation. **Kewu Huang:** Investigation. **Yahong Chen:** Investigation. **Yanfei Guo:** Investigation. **Guangliang Shan:** Methodology. **Ting Yang:** Supervision, Resources, Funding acquisition. **Chen Wang:** Supervision, Resources. **Jieping Lei:** Writing – review & editing, Writing – original draft, Supervision, Funding acquisition, Formal analysis, Data curation.

## Declaration of competing interest

The authors declare that they have no known competing financial interests or personal relationships that could have appeared to influence the work reported in this paper.

## Appendix Supplementary materials

Image, application 1
