# Supplementary material for: Prevalence of anxiety and depression and diagnostic utility of the CAT scale in spirometry-confirmed COPD patients: Findings from the Chinese Population Health and Multimorbidities Study
Source: Chin Med J Pulm Crit Care Med. 2026 Sep 7;4(3):278–85. doi: 10.1016/j.pccm.2026.08.003 (PMC13649495; doi:10.1016/j.pccm.2026.08.003)
Supplement: Supplementary file 1 [file mmc1.docx]

# Supplementary files

## Supplementary Table 1: Comparisons of the included versus excluded individuals in this study.

| **Variables** | **Study population (*n*=3641)** | **CAT or HADS missing (*n*=1267)** | **Statistics** | ***P* values** |
| --- | --- | --- | --- | --- |
| Age (years), median (IQR) | 61 (54, 68) | 62 (54, 69) | 3148302.00^*^ | 0.376 |
| Female, *n* (%) | 1327 (36.45) | 515 (40.65) | 7.08^†^ | 0.008 |
| BMI (kg/m^2^), median (IQR) | 23.59 (21.40, 25.89) | 23.55 (21.48, 26.08) | 3132614.50^*^ | 0.600 |
| Rural resident, *n* (%) | 1446 (39.71) | 413 (32.60) | 20.24^†^ | <0.001 |
| Annual mean PM_2.5_ exposure, *n* (%) |  |  |  |  |
| <50 μg/m^3^ | 224 (6.15) | 94 (7.42) | 2.90^†^ | 0.235 |
| 50–74 μg/m^3^ | 2281 (62.65) | 795 (62.75) |  |  |
| ≥75 μg/m^3^ | 1136 (31.20) | 378 (29.83) |  |  |
| Junior high school or above, *n* (%) | 2063 (56.66) | 776 (61.25) | 8.11^†^ | 0.004 |
| Currently married, *n* (%) | 3275 (89.95) | 1133 (89.42) | 0.01^†^ | 0.906 |
| Currently employed, *n* (%) | 2710 (74.43) | 968 (76.40) | 1.94^†^ | 0.163 |
| Family annual income (ten thousand yuan), median (IQR) | 2.00 (1.00, 5.00) | 3.00 (1.00, 5.00) | 2876344.50^*^ | 0.001 |
| Smoking status, *n* (%) |  |  |  |  |
| Never smoker | 1770 (48.61) | 709 (55.96) | 24.10^†^ | <0.001 |
| Ex-smoker | 479 (13.16) | 167 (13.18) |  |  |
| Current smoker | 1,392 (38.23) | 391 (30.86) |  |  |
| Smoking index (pack-years), median (IQR) | 0 (0, 31.00) | 0 (0, 24.25) | 2704008.00^*^ | <0.001 |
| Respiratory symptom, *n* (%) |  |  |  |  |
| Cough | 615 (16.89) | 190 (15.00) | 2.05^†^ | 0.152 |
| Sputum | 824 (22.63) | 237 (18.71) | 7.85^†^ | 0.005 |
| Wheeze | 686 (18.84) | 191 (15.07) | 8.40^†^ | 0.004 |
| Dyspnea | 694 (19.06) | 202 (15.94) | 5.70^†^ | 0.017 |
| Anxiety, *n* (%) | 403 (11.07) | 106 (8.37) | 7.38^†^ | 0.007 |
| Depression, *n* (%) | 518 (14.23) | 131 (10.34) | 12.38^†^ | <0.001 |
| Comorbidity, *n* (%) |  |  |  |  |
| Coronary heart disease | 113 (3.10) | 42 (3.31) | 0.44^†^ | 0.509 |
| Stroke | 45 (1.24) | 9 (0.71) | 1.96^†^ | 0.161 |
| Hypertension | 442 (12.14) | 147 (11.60) | 0.00^†^ | 0.948 |
| Diabetes | 134 (3.68) | 43 (3.39) | 0.03^†^ | 0.861 |
| Malignancy | 6 (0.16) | 2 (0.16) | NA^‡^ | 1.000 |
| Lung function indices, median (IQR) |  |  |  |  |
| FEV_1_ (L) | 2.05 (1.59, 2.57) | 2.10 (1.68, 2.60) | 3219779.00^*^ | 0.011 |
| FEV_1_%pred (%) | 81.91 (67.62, 94.21) | 84.80 (71.01, 97.03) | 3324192.00^*^ | <0.001 |
| FEV_1_/FVC (%) | 64.00 (56.41, 67.53) | 65.22 (60.23, 68.17) | 3389343.50^*^ | <0.001 |
| GOLD stage, *n* (%) |  |  |  |  |
| GOLD Ⅰ | 1,952 (53.61) | 767 (60.54) | 20.32^†^ | <0.001 |
| GOLD Ⅱ | 1,376 (37.79) | 422 (33.31) |  |  |
| GOLD Ⅲ–Ⅳ | 313 (8.60) | 78 (6.16) |  |  |

^*^*U* value (Mann-Whitney *U* test for continuous variable).

^†^*χ^2^* value (Chi-squared test for categorical variable).

^‡^“N/A” (not applicable), indicating Fisher’s exact test was applied and specific statistic was not applicable.

BMI, Body mass index; CAT, Chronic Obstructive Pulmonary Disease Assessment Test; FEV_1_, Forced expiratory volume in one second; FEV_1_%pred, Measured value of forced expiratory volume in one second to predictive value in percentage; FVC, Forced vital capacity; GOLD, Global Initiative for Chronic Obstructive Lung Disease; HADS, Hospital Anxiety and Depression Scale; IQR, Interquartile range; NA: Not Applicable; PM_2.5_: Particulate matter with a diameter less than 2.5 μm; SD: Standard Deviation.

## Supplementary Table 2: Comparisons of COPD patients with pulmonary- versus extra-pulmonary-dominant CAT subscales.

| **Variables** | **Spirometry-confirmed COPD patients (*N*=3641)** | | | |
| --- | --- | --- | --- | --- |
|  | **Q1234>Q5678 (*n*=2031)** | **Q1234≤Q5678 (*n*=1610)** | **Statistics** | ***P* values** |
| Age (years), median (IQR) | 61 (54, 68) | 61 (53, 68) | 2930577.00^*^ | 0.969 |
| Female, *n* (%) | 669 (32.94) | 658 (40.87) | 24.38^†^ | <0.001 |
| BMI (kg/m^2^), median (IQR) | 23.66 (21.45, 25.96) | 23.53 (21.33, 25.81) | 2904845.50^*^ | 0.392 |
| Rural resident, *n* (%) | 810 (39.88) | 636 (39.50) | 0.05^†^ | 0.817 |
| Annual mean PM_2.5_ exposure, *n* (%) |  |  |  |  |
| <50 μg/m^3^, | 108 (5.32) | 116 (7.20) | 8.06^†^ | 0.018 |
| 50–74 μg/m^3^, | 1,262 (62.14) | 1,019 (63.29) |  |  |
| ≥75 μg/m^3^, | 661 (32.55) | 475 (29.50) |  |  |
| Junior high school or above, *n* (%) | 1,128 (55.54) | 935 (58.07) | 2.35^†^ | 0.125 |
| Currently married, *n* (%) | 1,861 (91.63) | 1,414 (87.83) | 14.37^†^ | <0.001 |
| Currently employed, *n* (%) | 1,527 (75.18) | 1,183 (73.48) | 1.37^†^ | 0.241 |
| Family annual income (ten thousand yuan), median (IQR) | 2.00 (1.00, 5.00) | 2.00 (1.00, 5.00) | 2960400.00^*^ | 0.360 |
| Smoking status, *n* (%) |  |  |  |  |
| Never smoker | 883 (43.48) | 887 (55.09) | 57.38^†^ | <0.001 |
| Ex-smoker | 266 (13.10) | 213 (13.23) |  |  |
| Current smoker | 882 (43.43) | 510 (31.68) |  |  |
| Smoking index (pack-years), median (IQR) | 8.25 (0, 35.00) | 0 (0, 24.00) | 2504745.50^*^ | <0.001 |
| Respiratory symptom, *n* (%) |  |  |  |  |
| Cough | 465 (22.90) | 150 (9.32) | 118.30^†^ | <0.001 |
| Sputum | 619 (30.48) | 205 (12.73) | 160.27^†^ | <0.001 |
| Wheeze | 477 (23.49) | 209 (12.98) | 64.44^†^ | <0.001 |
| Dyspnea | 471 (23.19) | 223 (13.85) | 51.93^†^ | <0.001 |
| Anxiety, *n* (%) | 231 (11.37) | 172 (10.68) | 0.44^†^ | 0.510 |
| Depression, *n* (%) | 290 (14.28) | 228 (14.16) | 0.01^†^ | 0.920 |
| Comorbidity, *n* (%) |  |  |  |  |
| Coronary heart disease | 70 (3.45) | 43 (2.67) | 1.33^†^ | 0.248 |
| Stroke | 23 (1.13) | 22 (1.37) | 0.58^†^ | 0.448 |
| Hypertension | 250 (12.31) | 192 (11.93) | 0.00^†^ | 0.959 |
| Diabetes | 72 (3.55) | 62 (3.85) | 0.50^†^ | 0.482 |
| Malignancy | 3 (0.15) | 3 (0.19) | NA^‡^ | 1.000 |
| CAT |  |  |  |  |
| CAT total score (points), median (IQR) | 6 (3, 11) | 4 (0, 9) | 2479197.50^*^ | <0.001 |
| CAT total score ≥ 10, *n* (%) | 654 (32.20) | 387 (24.04) | 29.32^†^ | <0.001 |
| CAT total score categories, *n* (%) |  |  |  |  |
| 0–9 | 1,377 (67.80) | 1,223 (75.96) | 31.21^†^ | <0.001 |
| 10–19 | 494 (24.32) | 277 (17.20) |  |  |
| 20–29 | 139 (6.84) | 95 (5.90) |  |  |
| 30–40 | 21 (1.03) | 15 (0.93) |  |  |
| CAT Q1234 score (points), median (IQR) | 5 (3, 8) | 1 (0, 4) | 2029159.50^*^ | <0.001 |
| CAT Q5678 score (points), median (IQR) | 1 (0, 4) | 2 (0, 5) | 3166316.50^*^ | <0.001 |
| Lung function indices, median (IQR) |  |  |  |  |
| FEV_1_ (L) | 2.01 (1.56, 2.53) | 2.09 (1.65, 2.61) | 3041041.00^*^ | 0.001 |
| FEV_1_%pred (%) | 79.45 (64.60, 92.36) | 84.14 (71.63, 96.59) | 3172866.50^*^ | <0.001 |
| FEV_1_/FVC (%) | 62.99 (54.74, 67.06) | 65.12 (59.17, 67.89) | 3168210.00^*^ | <0.001 |
| GOLD stage, *n* (%) |  |  |  |  |
| GOLD Ⅰ | 996 (49.04) | 956 (59.38) | 44.46^†^ | <0.001 |
| GOLD Ⅱ | 824 (40.57) | 552 (34.29) |  |  |
| GOLD Ⅲ–Ⅳ | 211 (10.39) | 102 (6.34) |  |  |

^*^*U* value (Mann-Whitney *U* test for continuous variable).

^†^*χ^2^* value (Chi-squared test for categorical variable).

^‡^“N/A” (not applicable), indicating Fisher’s exact test was applied and specific statistic was not applicable.

CAT Q1234 are the first four questions in the CAT scale, assessing pulmonary symptoms (cough, phlegm/mucus, chest tightness, and breathlessness). CAT Q5678 are the remaining four questions in the CAT scale, assessing extra-pulmonary symptoms (activity, confidence, sleep, and energy).

BMI, Body mass index; CAT, Chronic Obstructive Pulmonary Disease Assessment Test; COPD, Chronic obstructive pulmonary disease; FEV_1_, Forced expiratory volume in one second; FEV_1_%pred, Measured value of forced expiratory volume in one second to predictive value in percentage; FVC, Forced vital capacity; GOLD, Global Initiative for Chronic Obstructive Lung Disease; IQR, Interquartile range; NA, Not applicable; PM_2.5_, Particulate matter with a diameter less than 2.5 μm; SD: Standard deviation.

## Supplementary Table 3: Associations of CAT total score (excluded the sixth [confidence] and the eighth [energy] items) and other influencing factors with comorbidity of anxiety or depression in COPD patients, respectively.

| **Associated factors** | **Anxiety** | |  | **Depression** | |
| --- | --- | --- | --- | --- | --- |
|  | **OR (95% CI)** | ***P* value** |  | **OR (95% CI)** | ***P* value** |
| Age (years) | 0.989 (0.977–1.001) | 0.070 |  | 1.001 (0.990–1.012) | 0.906 |
| Female | 1.355 (1.017–1.806) | 0.038 |  | 1.105 (0.856–1.428) | 0.443 |
| BMI (kg/m^2^) | 0.993 (0.959–1.029) | 0.713 |  | 0.988 (0.957–1.019) | 0.438 |
| Rural resident | 1.641 (1.264–2.129) | <0.001 |  | 1.758 (1.392–2.220) | <0.001 |
| Annual mean PM_2.5_ exposure |  |  |  |  |  |
| <50 μg/m^3^ | Ref. |  |  | Ref. |  |
| 50–74 μg/m^3^ | 1.378 (0.845–2.246) | 0.199 |  | 1.197 (0.775–1.848) | 0.418 |
| ≥75 μg/m^3^ | 1.010 (0.590–1.729) | 0.972 |  | 0.968 (0.602–1.559) | 0.895 |
| Junior high school or above | 1.006 (0.765–1.324) | 0.966 |  | 0.821 (0.643–1.049) | 0.114 |
| Currently employed | 0.684 (0.522–0.898) | 0.006 |  | 0.765 (0.599–0.977) | 0.032 |
| Family annual income (ten thousand yuan) | 0.910 (0.860–0.963) | 0.001 |  | 0.959 (0.918–1.001) | 0.055 |
| Smoking index (pack-years) | 1.000 (0.994–1.006) | 0.944 |  | 0.994 (0.988–0.999) | 0.031 |
| Coronary heart disease | 1.202 (0.677–2.134) | 0.531 |  | 1.225 (0.733–2.048) | 0.439 |
| Stroke | 1.117 (0.446–2.799) | 0.813 |  | 1.942 (0.944–3.996) | 0.072 |
| Hypertension | 0.857 (0.594–1.238) | 0.411 |  | 0.865 (0.628–1.191) | 0.373 |
| Diabetes | 0.714 (0.368–1.388) | 0.321 |  | 0.883 (0.513–1.521) | 0.654 |
| Malignancy | 1.030 (0.114–9.348) | 0.979 |  | NA | NA |
| CAT total score excluded item 6 & 8^*^ | 1.080 (1.058–1.103) | <0.001 |  | 1.068 (1.048–1.088) | <0.001 |
| GOLD stage |  |  |  |  |  |
| GOLD Ⅰ | Ref. |  |  | Ref. |  |
| GOLD Ⅱ | 0.913 (0.700–1.191) | 0.502 |  | 0.957 (0.755–1.213) | 0.718 |
| GOLD Ⅲ-Ⅳ | 1.095 (0.735–1.631) | 0.656 |  | 1.225 (0.857–1.752) | 0.266 |

BMI: Body mass index; CAT: Chronic Obstructive Pulmonary Disease Assessment Test; CI: Confidence interval; COPD: Chronic obstructive pulmonary disease; GOLD: Global Initiative for Chronic Obstructive Lung Disease; NA: Not applicable; OR: Odds ratio; PM_2.5_: Particulate matter with a diameter less than 2.5 μm; Ref.: Reference group.

^*^**The sixth item of the CAT scale:** I am confident leaving my home despite my lung condition. / I am not at all confident leaving my home because of my lung condition.; **the eighth item of the CAT scale:** I have lots of energy. / I have no energy at all.

## Supplementary Table 4: Associations of CAT total score, extra-pulmonary-dominant CAT subscale, and other influencing factors with comorbidity of anxiety or depression in COPD patients, respectively.

| **Associated factors** | **Anxiety** | |  | **Depression** | |
| --- | --- | --- | --- | --- | --- |
|  | **OR (95% CI)** | ***P* values** |  | **OR (95% CI)** | ***P* values** |
| Age (years) | 0.988 (0.976–1.000) | 0.058 |  | 1.000 (0.989–1.011) | 0.997 |
| Female | 1.353 (1.015–1.804) | 0.039 |  | 1.098 (0.849–1.419) | 0.476 |
| BMI (kg/m^2^) | 0.995 (0.961–1.031) | 0.778 |  | 0.989 (0.958–1.021) | 0.505 |
| Rural resident | 1.648 (1.270–2.139) | <0.001 |  | 1.772 (1.403–2.239) | <0.001 |
| Annual mean PM_2.5_ exposure |  |  |  |  |  |
| <50 μg/m^3^ | Ref. |  |  | Ref. |  |
| 50–74 μg/m^3^ | 1.427 (0.872–2.336) | 0.157 |  | 1.257 (0.811–1.949) | 0.306 |
| ≥75 μg/m^3^ | 1.056 (0.614–1.815) | 0.845 |  | 1.022 (0.633–1.652) | 0.928 |
| Junior high school or above | 1.005 (0.764–1.322) | 0.972 |  | 0.821 (0.642–1.049) | 0.114 |
| Currently employed | 0.693 (0.528–0.909) | 0.008 |  | 0.776 (0.607–0.993) | 0.044 |
| Family annual income (ten thousand yuan) | 0.910 (0.860–0.963) | 0.001 |  | 0.959 (0.919–1.001) | 0.057 |
| Smoking index (pack-years) | 1.000 (0.994–1.006) | 0.998 |  | 0.994 (0.989–1.000) | 0.038 |
| Coronary heart disease | 1.191 (0.670–2.118) | 0.551 |  | 1.206 (0.719–2.023) | 0.478 |
| Stroke | 1.056 (0.419–2.663) | 0.908 |  | 1.834 (0.885–3.799) | 0.103 |
| Hypertension | 0.852 (0.590–1.230) | 0.392 |  | 0.857 (0.622–1.181) | 0.346 |
| Diabetes | 0.717 (0.369–1.393) | 0.326 |  | 0.885 (0.514–1.525) | 0.660 |
| Malignancy | 0.963 (0.107–8.693) | 0.974 |  | NA | NA |
| CAT total score | 1.063 (1.046–1.080) | <0.001 |  | 1.057 (1.041–1.072) | <0.001 |
| CAT Q5678 ≥ Q1234 score | 1.040 (0.812–1.333) | 0.754 |  | 1.129 (0.905–1.409) | 0.283 |
| GOLD stage |  |  |  |  |  |
| GOLD Ⅰ | Ref. |  |  | Ref. |  |
| GOLD Ⅱ | 0.929 (0.712–1.212) | 0.587 |  | 0.973 (0.767–1.234) | 0.821 |
| GOLD Ⅲ–Ⅳ | 1.117 (0.750–1.665) | 0.586 |  | 1.235 (0.863–1.768) | 0.248 |

CAT Q1234 are the first four questions in the CAT scale, assessing pulmonary symptoms (cough, phlegm/mucus, chest tightness, and breathlessness). CAT Q5678 are the remaining four questions in the CAT scale, assessing extra-pulmonary symptoms (activity, confidence, sleep, and energy).

BMI, Body mass index; CAT, Chronic Obstructive Pulmonary Disease Assessment Test; CI, Confidence interval; COPD, Chronic obstructive pulmonary disease; GOLD, Global Initiative for Chronic Obstructive Lung Disease; NA, Not applicable; OR, Odds ratio; PM_2.5_, Particulate matter with a diameter less than 2.5 μm; SD, Standard deviation; Ref.: Reference group.

## Supplementary Table 5: CAT total score and the diagnostic performance for anxiety assessment in COPD patients.

| **CAT total score** | **Specificity (%)** | **Sensitivity (%)** | **Youden index** |
| --- | --- | --- | --- |
| 9 | 69.117 | 51.861 | 0.210 |
| 8 | 62.786 | 57.568 | 0.204 |
| 10 | 73.626 | 46.402 | 0.200 |
| 11 | 77.795 | 42.184 | 0.200 |
| 7 | 57.690 | 62.283 | 0.200 |
| 6 | 52.224 | 66.005 | 0.182 |
| 12 | 80.420 | 37.717 | 0.181 |
| 14 | 85.207 | 32.010 | 0.172 |
| 13 | 82.860 | 34.243 | 0.171 |
| 5 | 45.244 | 70.968 | 0.162 |
| 15 | 87.091 | 28.040 | 0.151 |
| 16 | 88.697 | 26.303 | 0.150 |
| 4 | 38.419 | 75.682 | 0.141 |
| 17 | 90.550 | 23.325 | 0.139 |
| 3 | 31.347 | 82.382 | 0.137 |
| 18 | 91.847 | 20.844 | 0.127 |
| 19 | 92.990 | 18.362 | 0.114 |
| 20 | 93.792 | 17.122 | 0.109 |
| 21 | 94.719 | 15.136 | 0.099 |
| 2 | 21.433 | 87.841 | 0.093 |
| 22 | 95.584 | 13.151 | 0.087 |
| 1 | 15.627 | 93.052 | 0.087 |
| 24 | 96.881 | 10.670 | 0.076 |
| 23 | 96.325 | 11.166 | 0.075 |
| 25 | 97.653 | 8.685 | 0.063 |
| 26 | 98.085 | 7.692 | 0.058 |
| 27 | 98.456 | 5.211 | 0.037 |
| 28 | 98.703 | 4.218 | 0.029 |
| 29 | 99.043 | 3.226 | 0.023 |
| 30 | 99.197 | 2.481 | 0.017 |
| 31 | 99.413 | 1.737 | 0.012 |
| 32 | 99.475 | 1.489 | 0.010 |
| 33 | 99.660 | 0.744 | 0.004 |
| 34 | 99.815 | 0.496 | 0.003 |
| 35 | 99.815 | 0.496 | 0.003 |
| 36 | 99.815 | 0.496 | 0.003 |
| 39 | 99.969 | 0.000 | -0.000 |
| 40 | 99.969 | 0.000 | -0.000 |
| 37 | 99.907 | 0.000 | -0.001 |
| 38 | 99.907 | 0.000 | -0.001 |

CAT, Chronic Obstructive Pulmonary Disease Assessment Test; COPD, Chronic obstructive pulmonary disease.

## Supplementary Table 6: CAT total score and the diagnostic performance for anxiety assessment in COPD patients in rural or urban area, respectively.

| **Rural area** | | | |  | **Urban area** | | | |
| --- | --- | --- | --- | --- | --- | --- | --- | --- |
| **CAT total score** | **Specificity (%)** | **Sensitivity (%)** | **Youden index** |  | **CAT total score** | **Specificity (%)** | **Sensitivity (%)** | **Youden index** |
| 7 | 58.900 | 61.429 | 0.203 |  | 11 | 77.522 | 46.114 | 0.236 |
| 8 | 63.835 | 55.714 | 0.195 |  | 9 | 68.332 | 54.922 | 0.233 |
| 9 | 70.388 | 49.048 | 0.194 |  | 10 | 73.127 | 49.223 | 0.223 |
| 10 | 74.434 | 43.810 | 0.182 |  | 8 | 62.138 | 59.585 | 0.217 |
| 6 | 53.074 | 64.762 | 0.178 |  | 14 | 85.714 | 35.751 | 0.215 |
| 5 | 46.521 | 70.952 | 0.175 |  | 13 | 82.967 | 37.824 | 0.208 |
| 11 | 78.236 | 38.571 | 0.168 |  | 12 | 80.470 | 39.896 | 0.204 |
| 12 | 80.340 | 35.714 | 0.161 |  | 7 | 56.943 | 63.212 | 0.202 |
| 3 | 32.201 | 82.381 | 0.146 |  | 15 | 87.812 | 32.124 | 0.199 |
| 4 | 39.239 | 75.238 | 0.145 |  | 16 | 89.461 | 30.052 | 0.195 |
| 13 | 82.686 | 30.952 | 0.136 |  | 6 | 51.698 | 67.358 | 0.191 |
| 14 | 84.385 | 28.571 | 0.130 |  | 17 | 91.259 | 26.943 | 0.182 |
| 16 | 87.460 | 22.857 | 0.103 |  | 5 | 44.456 | 70.984 | 0.154 |
| 15 | 85.922 | 24.286 | 0.102 |  | 18 | 92.507 | 22.798 | 0.153 |
| 19 | 92.233 | 17.619 | 0.099 |  | 4 | 37.912 | 76.166 | 0.141 |
| 18 | 90.777 | 19.048 | 0.098 |  | 3 | 30.819 | 82.383 | 0.132 |
| 17 | 89.401 | 20.000 | 0.094 |  | 20 | 94.106 | 18.653 | 0.128 |
| 20 | 93.285 | 15.714 | 0.090 |  | 19 | 93.457 | 19.171 | 0.126 |
| 21 | 94.337 | 13.810 | 0.081 |  | 2 | 20.979 | 90.674 | 0.117 |
| 2 | 22.168 | 85.238 | 0.074 |  | 21 | 94.955 | 16.580 | 0.115 |
| 22 | 95.793 | 10.952 | 0.067 |  | 1 | 15.784 | 95.337 | 0.111 |
| 1 | 15.372 | 90.952 | 0.063 |  | 22 | 95.455 | 15.544 | 0.110 |
| 23 | 96.602 | 9.048 | 0.056 |  | 24 | 96.803 | 12.953 | 0.098 |
| 24 | 97.006 | 8.571 | 0.056 |  | 23 | 96.154 | 13.472 | 0.096 |
| 25 | 97.896 | 5.714 | 0.036 |  | 25 | 97.503 | 11.917 | 0.094 |
| 26 | 98.139 | 5.238 | 0.034 |  | 26 | 98.052 | 10.363 | 0.084 |
| 27 | 98.382 | 3.333 | 0.017 |  | 27 | 98.502 | 7.254 | 0.058 |
| 28 | 98.786 | 2.857 | 0.016 |  | 28 | 98.651 | 5.699 | 0.044 |
| 29 | 98.948 | 2.381 | 0.013 |  | 29 | 99.101 | 4.145 | 0.032 |
| 34 | 99.838 | 0.952 | 0.008 |  | 30 | 99.201 | 3.627 | 0.028 |
| 35 | 99.838 | 0.952 | 0.008 |  | 31 | 99.351 | 2.591 | 0.019 |
| 36 | 99.838 | 0.952 | 0.008 |  | 32 | 99.451 | 2.073 | 0.015 |
| 33 | 99.676 | 0.952 | 0.006 |  | 33 | 99.650 | 0.518 | 0.002 |
| 30 | 99.191 | 1.429 | 0.006 |  | 39 | 99.950 | 0.000 | -0.000 |
| 31 | 99.515 | 0.952 | 0.005 |  | 40 | 99.950 | 0.000 | -0.000 |
| 32 | 99.515 | 0.952 | 0.005 |  | 37 | 99.900 | 0.000 | -0.001 |
| 39 | 100.000 | 0.000 | 0.000 |  | 38 | 99.900 | 0.000 | -0.001 |
| 40 | 100.000 | 0.000 | 0.000 |  | 34 | 99.800 | 0.000 | -0.002 |
| 37 | 99.919 | 0.000 | -0.001 |  | 35 | 99.800 | 0.000 | -0.002 |
| 38 | 99.919 | 0.000 | -0.001 |  | 36 | 99.800 | 0.000 | -0.002 |

CAT, Chronic Obstructive Pulmonary Disease Assessment Test; COPD, Chronic obstructive pulmonary disease.

## Supplementary Table 7: CAT pulmonary and extra-pulmonary component scores and the diagnostic performance for anxiety assessment in COPD patients.

| **CAT Q1234 score** | **Specificity (%)** | **Sensitivity (%)** | **Youden index** |  | **CAT Q5678 score** | **Specificity (%)** | **Sensitivity (%)** | **Youden index** |
| --- | --- | --- | --- | --- | --- | --- | --- | --- |
| 5 | 63.650 | 53.846 | 0.175 |  | 4 | 68.159 | 52.109 | 0.203 |
| 6 | 71.309 | 45.906 | 0.172 |  | 3 | 59.728 | 59.305 | 0.190 |
| 4 | 51.266 | 65.509 | 0.168 |  | 5 | 77.733 | 40.447 | 0.182 |
| 7 | 78.135 | 37.469 | 0.156 |  | 2 | 48.054 | 68.983 | 0.170 |
| 8 | 82.520 | 32.754 | 0.153 |  | 6 | 82.922 | 33.747 | 0.167 |
| 3 | 40.766 | 72.953 | 0.137 |  | 7 | 86.875 | 29.777 | 0.167 |
| 9 | 86.566 | 26.799 | 0.134 |  | 8 | 89.469 | 25.806 | 0.153 |
| 10 | 89.284 | 22.829 | 0.121 |  | 1 | 37.276 | 77.916 | 0.152 |
| 2 | 28.567 | 82.878 | 0.114 |  | 9 | 92.495 | 18.362 | 0.109 |
| 11 | 91.970 | 19.107 | 0.111 |  | 10 | 94.503 | 15.633 | 0.101 |
| 1 | 20.723 | 89.330 | 0.101 |  | 11 | 95.954 | 12.407 | 0.084 |
| 12 | 93.885 | 15.633 | 0.095 |  | 12 | 97.004 | 9.926 | 0.069 |
| 13 | 95.985 | 11.663 | 0.076 |  | 13 | 98.085 | 6.700 | 0.048 |
| 14 | 97.004 | 8.685 | 0.057 |  | 14 | 98.610 | 3.970 | 0.026 |
| 15 | 97.869 | 5.955 | 0.038 |  | 15 | 99.074 | 1.737 | 0.008 |
| 16 | 98.672 | 4.467 | 0.031 |  | 16 | 99.259 | 1.241 | 0.005 |
| 17 | 99.321 | 2.481 | 0.018 |  | 17 | 99.722 | 0.744 | 0.005 |
| 18 | 99.475 | 1.985 | 0.015 |  | 19 | 99.907 | 0.248 | 0.002 |
| 19 | 99.753 | 1.241 | 0.010 |  | 18 | 99.815 | 0.248 | 0.001 |
| 20 | 99.846 | 0.496 | 0.003 |  | 20 | 99.938 | 0.000 | -0.001 |

CAT Q1234 are the first four questions in the CAT scale, assessing pulmonary symptoms (cough, phlegm/mucus, chest tightness, and breathlessness). CAT Q5678 are the remaining four questions in the CAT scale, assessing extra-pulmonary symptoms (activity, confidence, sleep, and energy).

CAT, Chronic Obstructive Pulmonary Disease Assessment Test; COPD, Chronic obstructive pulmonary disease.

## Supplementary Table 8: CAT total score and the diagnostic performance for depression assessment in COPD patients.

| **CAT total score** | **Specificity (%)** | **Sensitivity (%)** | **Youden index** |
| --- | --- | --- | --- |
| 7 | 58.501 | 62.741 | 0.212 |
| 8 | 63.529 | 57.529 | 0.211 |
| 6 | 53.154 | 67.568 | 0.207 |
| 9 | 69.677 | 50.579 | 0.203 |
| 10 | 74.224 | 45.560 | 0.198 |
| 11 | 78.098 | 39.575 | 0.177 |
| 5 | 45.917 | 71.429 | 0.173 |
| 12 | 80.660 | 35.135 | 0.158 |
| 4 | 39.065 | 76.448 | 0.155 |
| 13 | 83.093 | 31.853 | 0.149 |
| 14 | 85.207 | 28.185 | 0.134 |
| 3 | 31.668 | 81.274 | 0.129 |
| 15 | 87.128 | 24.903 | 0.120 |
| 16 | 88.697 | 22.973 | 0.117 |
| 17 | 90.522 | 20.077 | 0.106 |
| 2 | 21.806 | 88.031 | 0.098 |
| 19 | 93.020 | 16.023 | 0.090 |
| 18 | 91.707 | 17.181 | 0.089 |
| 1 | 15.882 | 92.664 | 0.085 |
| 20 | 93.756 | 14.479 | 0.082 |
| 21 | 94.621 | 12.355 | 0.070 |
| 22 | 95.517 | 10.811 | 0.063 |
| 24 | 96.798 | 8.494 | 0.053 |
| 23 | 96.190 | 8.687 | 0.049 |
| 25 | 97.534 | 6.564 | 0.041 |
| 26 | 98.015 | 5.985 | 0.040 |
| 28 | 98.719 | 3.668 | 0.024 |
| 27 | 98.367 | 3.861 | 0.022 |
| 29 | 99.103 | 3.089 | 0.022 |
| 30 | 99.296 | 2.703 | 0.020 |
| 32 | 99.648 | 2.317 | 0.020 |
| 31 | 99.552 | 2.317 | 0.019 |
| 33 | 99.744 | 1.158 | 0.009 |
| 34 | 99.840 | 0.579 | 0.004 |
| 35 | 99.840 | 0.579 | 0.004 |
| 36 | 99.840 | 0.579 | 0.004 |
| 39 | 99.968 | 0.000 | -0.000 |
| 40 | 99.968 | 0.000 | -0.000 |
| 37 | 99.904 | 0.000 | -0.001 |
| 38 | 99.904 | 0.000 | -0.001 |

CAT, Chronic Obstructive Pulmonary Disease Assessment Test; COPD, Chronic obstructive pulmonary disease.

## Supplementary Table 9: CAT total score and the diagnostic performance for depression assessment in COPD patients in rural or urban area, respectively.

| **Rural area** | | | |  | **Urban area** | | | |
| --- | --- | --- | --- | --- | --- | --- | --- | --- |
| **CAT total score** | **Specificity (%)** | **Sensitivity (%)** | **Youden index** |  | **CAT total score** | **Specificity (%)** | **Sensitivity (%)** | **Youden index** |
| 7 | 59.745 | 60.517 | 0.203 |  | 10 | 73.819 | 49.798 | 0.236 |
| 6 | 54.213 | 65.683 | 0.199 |  | 8 | 62.885 | 60.729 | 0.236 |
| 8 | 64.596 | 54.613 | 0.192 |  | 7 | 57.752 | 65.182 | 0.229 |
| 9 | 71.064 | 47.601 | 0.187 |  | 9 | 68.840 | 53.846 | 0.227 |
| 5 | 47.319 | 70.480 | 0.178 |  | 6 | 52.515 | 69.636 | 0.222 |
| 10 | 74.894 | 41.697 | 0.166 |  | 11 | 77.823 | 43.320 | 0.211 |
| 4 | 40.000 | 75.277 | 0.153 |  | 13 | 83.162 | 34.818 | 0.180 |
| 11 | 78.553 | 36.162 | 0.147 |  | 12 | 80.698 | 37.247 | 0.179 |
| 12 | 80.596 | 33.210 | 0.138 |  | 5 | 45.072 | 72.470 | 0.175 |
| 13 | 82.979 | 29.151 | 0.121 |  | 14 | 85.678 | 30.769 | 0.164 |
| 3 | 32.340 | 79.705 | 0.120 |  | 4 | 38.501 | 77.733 | 0.162 |
| 14 | 84.426 | 25.830 | 0.103 |  | 15 | 87.782 | 27.530 | 0.153 |
| 15 | 86.043 | 22.509 | 0.086 |  | 16 | 89.425 | 25.506 | 0.149 |
| 16 | 87.489 | 20.664 | 0.082 |  | 17 | 91.273 | 23.077 | 0.144 |
| 2 | 22.468 | 84.871 | 0.073 |  | 3 | 31.263 | 82.996 | 0.143 |
| 19 | 92.085 | 14.760 | 0.068 |  | 2 | 21.407 | 91.498 | 0.129 |
| 17 | 89.277 | 17.343 | 0.066 |  | 18 | 92.454 | 19.028 | 0.115 |
| 1 | 15.574 | 90.406 | 0.060 |  | 1 | 16.068 | 95.142 | 0.112 |
| 18 | 90.468 | 15.498 | 0.060 |  | 19 | 93.583 | 17.409 | 0.110 |
| 20 | 93.021 | 12.546 | 0.056 |  | 20 | 94.199 | 16.599 | 0.108 |
| 21 | 94.128 | 11.070 | 0.052 |  | 21 | 94.918 | 13.765 | 0.087 |
| 22 | 95.660 | 8.856 | 0.045 |  | 22 | 95.431 | 12.955 | 0.084 |
| 24 | 96.851 | 6.642 | 0.035 |  | 26 | 98.152 | 9.312 | 0.075 |
| 23 | 96.426 | 7.011 | 0.034 |  | 24 | 96.766 | 10.526 | 0.073 |
| 25 | 97.617 | 3.690 | 0.013 |  | 25 | 97.485 | 9.717 | 0.072 |
| 33 | 99.745 | 1.107 | 0.009 |  | 23 | 96.047 | 10.526 | 0.066 |
| 26 | 97.787 | 2.952 | 0.007 |  | 27 | 98.563 | 6.478 | 0.050 |
| 31 | 99.574 | 1.107 | 0.007 |  | 28 | 98.819 | 6.073 | 0.049 |
| 32 | 99.574 | 1.107 | 0.007 |  | 29 | 99.281 | 4.858 | 0.041 |
| 34 | 99.830 | 0.738 | 0.006 |  | 30 | 99.384 | 4.453 | 0.038 |
| 35 | 99.830 | 0.738 | 0.006 |  | 32 | 99.692 | 3.644 | 0.033 |
| 36 | 99.830 | 0.738 | 0.006 |  | 31 | 99.538 | 3.644 | 0.032 |
| 29 | 98.809 | 1.476 | 0.003 |  | 33 | 99.743 | 1.215 | 0.010 |
| 30 | 99.149 | 1.107 | 0.003 |  | 34 | 99.846 | 0.405 | 0.003 |
| 28 | 98.553 | 1.476 | 0.000 |  | 35 | 99.846 | 0.405 | 0.003 |
| 39 | 100.000 | 0.000 | 0.000 |  | 36 | 99.846 | 0.405 | 0.003 |
| 40 | 100.000 | 0.000 | 0.000 |  | 39 | 99.949 | 0.000 | -0.001 |
| 37 | 99.915 | 0.000 | -0.001 |  | 40 | 99.949 | 0.000 | -0.001 |
| 38 | 99.915 | 0.000 | -0.001 |  | 37 | 99.897 | 0.000 | -0.001 |
| 27 | 98.043 | 1.476 | -0.005 |  | 38 | 99.897 | 0.000 | -0.001 |

CAT, Chronic Obstructive Pulmonary Disease Assessment Test; COPD, Chronic obstructive pulmonary disease.

## Supplementary Table 10: CAT pulmonary and extra-pulmonary component scores and the diagnostic performance for depression assessment in COPD patients.

| **CAT Q1234 score** | **Specificity (%)** | **Sensitivity (%)** | **Youden index** |  | **CAT Q5678 score** | **Specificity (%)** | **Sensitivity (%)** | **Youden index** |
| --- | --- | --- | --- | --- | --- | --- | --- | --- |
| 5 | 64.233 | 53.475 | 0.177 |  | 4 | 68.780 | 51.351 | 0.201 |
| 6 | 71.790 | 44.981 | 0.168 |  | 3 | 60.423 | 59.266 | 0.197 |
| 4 | 51.777 | 64.865 | 0.166 |  | 2 | 48.831 | 69.884 | 0.187 |
| 7 | 78.450 | 35.907 | 0.144 |  | 5 | 78.130 | 38.803 | 0.169 |
| 8 | 82.709 | 30.502 | 0.132 |  | 1 | 37.848 | 77.992 | 0.158 |
| 3 | 41.114 | 72.008 | 0.131 |  | 6 | 83.285 | 32.239 | 0.155 |
| 2 | 28.818 | 81.853 | 0.107 |  | 7 | 87.160 | 27.799 | 0.150 |
| 1 | 21.069 | 89.189 | 0.103 |  | 8 | 89.657 | 23.552 | 0.132 |
| 9 | 86.519 | 23.552 | 0.101 |  | 9 | 92.571 | 16.409 | 0.090 |
| 10 | 89.177 | 19.498 | 0.087 |  | 10 | 94.396 | 12.741 | 0.071 |
| 12 | 93.788 | 12.934 | 0.067 |  | 11 | 95.965 | 10.618 | 0.066 |
| 11 | 91.675 | 14.865 | 0.065 |  | 12 | 97.054 | 8.687 | 0.057 |
| 13 | 95.805 | 8.880 | 0.047 |  | 13 | 98.111 | 5.792 | 0.039 |
| 14 | 96.926 | 6.950 | 0.039 |  | 14 | 98.751 | 4.247 | 0.030 |
| 15 | 97.855 | 5.019 | 0.029 |  | 15 | 99.135 | 1.931 | 0.011 |
| 16 | 98.655 | 3.668 | 0.023 |  | 16 | 99.328 | 1.544 | 0.009 |
| 17 | 99.360 | 2.317 | 0.017 |  | 17 | 99.744 | 0.772 | 0.005 |
| 18 | 99.520 | 1.931 | 0.015 |  | 19 | 99.904 | 0.193 | 0.001 |
| 19 | 99.776 | 1.158 | 0.009 |  | 18 | 99.808 | 0.193 | 0.000 |
| 20 | 99.840 | 0.386 | 0.002 |  | 20 | 99.936 | 0.000 | -0.001 |

CAT Q1234 are the first four questions in the CAT scale, assessing pulmonary symptoms (cough, phlegm/mucus, chest tightness, and breathlessness). CAT Q5678 are the remaining four questions in the CAT scale, assessing extra-pulmonary symptoms (activity, confidence, sleep, and energy).

CAT, Chronic Obstructive Pulmonary Disease Assessment Test; COPD, Chronic obstructive pulmonary disease.

## Supplementary File

We used the following methods to assess the fitness of multivariable logistic regression models in this study:

(1) First, the main model (Model 1) consisted of 16 variables, and Model 2 and Model 3 both had 17 variables, respectively. In the included 3641 COPD patients, 403 and 518 patients were identified as having anxiety and depression, respectively. In the main model, after excluding the individuals with missing information on the analytical variables, the number of patients having anxiety was 321, and EPV (Events Per Variable) was 321/16 = 20; the number of patients having depression was 419, EPV was 419/16 = 26. In Model 2 and Model 3, EPV was 321/17 = 19 for anxiety analysis, and EPV was 419/17 = 25 for depression analysis, respectively.

(2) We performed the following additional analyses based on the main multivariable logistic regression model for anxiety:

① Calculated the area under the receiver operating characteristic (ROC) curve: C index=0.6851.

② Performed the Hosmer-Lemeshow goodness-of-fit test: *P* value=0.0262.

③ Randomly split the data into training set and validation set at a 7:3 ratio, constructed two main models and calculated the AUCs respectively: training set C index=0.6852, validation set C index=0.6856, the difference was 0.0004.

④ Calculated the predicted probability and actual occurrence rate of the outcome event (i.e. prevalence of anxiety), and drew the calibration plot (as shown below). The calibration curve fitted well.

⑤ Performed collinearity analysis and found that all variance inflation factors of the analytical variables were < 1.5, which indicated no significant collinearity between variables in the main model.

Similar findings were observed in Model 2 and Model 3.

(3) We performed following additional analyses based on the main multivariable logistic regression model for depression:

① Calculated the AUC: C index=0.6810.

② Performed the Hosmer-Lemeshow goodness-of-fit test: *P* value=0.2247.

③ Randomly split the data into training set and validation set at a 7:3 ratio, constructed two main models and calculated the AUCs respectively: training set C index=0.6831, validation set C index=0.6830, the difference was 0.0001.

④ Calculated the predicted probability and actual occurrence rate of the outcome event (i.e. prevalence of depression), and drew the calibration plot (as shown below). The calibration curve fitted well.

⑤ Performed collinearity analysis and found that all variance inflation factors of the analytical variables were < 1.5, which indicated no significant collinearity between variables in the main model.

Similar findings were observed in Model 2 and Model 3.

(4) In summary, the multivariable models constructed for anxiety and depression assessment in COPD patients showed good fit in this study, however, external validation is warranted in the future.
